# Coupling of Limit Switch Sensors and Stepper Motors with Acoustic Feedback for Positioning of a Cartesian Robot End Effector in the Study of Musical Instruments

**DOI:** 10.3390/s25061709

**Published:** 2025-03-10

**Authors:** Daniel Tokarczyk, Jan Jasiński, Marek Pluta, Jerzy Wiciak

**Affiliations:** AGH University of Krakow, Department of Mechanics and Vibroacoustics, av. Mickiewicza 30, 30-059 Krakow, Poland; danielt@agh.edu.pl (D.T.); jjasinsk@agh.edu.pl (J.J.); pluta@agh.edu.pl (M.P.)

**Keywords:** string excitation, string vibration, robot, sensors, musical acoustics, robotic musicianship, musical instruments, guitar, chordophones

## Abstract

This article discusses the innovative application of a Cartesian robot manipulator with acoustic feedback for calibration and precise positioning of a string-excitation element in investigating stringed instruments. It describes an experiment in which an acoustic guitar string is automatically excited with different guitar picks. The robot’s end effector positioning system utilizes limit switches, acting as a mechanical sensor, which provides feedback to the linear actuators that are equipped with stepper motors. The authors detail the research challenges they faced and propose a positioning algorithm that makes use of a microphone as an acoustic sensor, improving the calibration of the end effector’s position.

## 1. Introduction

Musical instruments are designed with two self-evident assumptions: their purpose is to produce sound characterized by specific qualities, and they are to be operated by humans. These assumptions profoundly impact the forms of instruments—in particular, their geometric and material properties. Acoustic and ergonomic considerations often lead to complex shapes with curvatures, and the role of a particular instrument’s element in the process of sound production often forbids restraining its vibrations. This seriously limits possible ways an instrument can be held while producing sound. As a consequence, it is difficult to design laboratory stands for studying instruments’ properties under normal operating conditions with repeatable results, because they need to follow human grip patterns, which are inherently unstable.

The problem can be solved in several ways. One tactic involves prioritizing repeatability and stability over normal operating conditions. This involves disassembling the instrument, securing its components, and examining each part individually. This makes data collection easier, and it has been a common strategy in previous guitar research, to gain insights into the vibrational properties of guitar top plates, for example, with a free edge and without braces [1], or with fixed ribs but without a back plate [2]. However, this method has its limitations as the vibrational characteristics of the components may differ in a fully assembled instrument, due to varying boundary conditions and the interconnected vibrations of the adjacent parts [3]. A second approach is to study an entire instrument under conditions as close as possible to natural—that is, played by a human and evaluated by a human [4]. This approach, coupled with statistical analysis, can yield reliable information on the audibility of generalized change in the instrument sound timbre, allowing us to determine whether listeners hear the difference between the sound of two instrument specimens, or if they hear the result of introducing some significant change to the instrument. However, collecting more specific data can be challenging, due to the numerous uncontrollable or unquantifiable variables. The third strategy mitigates the human factor by studying the instrument in its entirety, mounted on a fixed stand, and excited mechanically. This allows for the acquisition of more accurate data and research on, for example, the influence on guitar sound characteristics of the stability of selected quantities [5], sound hole geometry [6], or tonewood choice [7], or even of the vibration acceleration spectrum in a cello [8]. This approach can be more precise and repeatable than human playing/evaluation, but, due to the need to fix an entire instrument without affecting its sound, it is less precise than a study of extracted instrument elements. Being able to perform only a single type of simple instrument excitation, the third approach is also less universal than the second approach.

Simple excitation mechanisms can be replaced by robots. While not as versatile as a human, a robot can precisely perform complex movement patterns. Using a robot may allow us to collect accurate data regarding an instrument or a signal it produces in scenarios closer to the real conditions of an instrument’s operation. Moreover, various study scenarios may be programmed, repeated, and modified at a request.

Musical robots have been used mainly for performative purposes [9,10,11], but nowadays, due to the technological possibilities available to researchers, it is possible to design more sophisticated experiments on musical instruments. For that purpose, the usage of musical robots is becoming more and more popular [12,13,14], although most designs focus on the research of control systems and human–robot interaction [15,16,17,18].

The choice of a particular approach to the issue of automated instrument excitation depends on the character and purpose of the study. The first type of research scenario is the study of an instrument itself, in order to establish relations between its material or geometric properties and the qualities of the sound that it produces [1,2,4]. In this case, the priority is precision, repeatability, and the ability to test and compare multiple specimens. Therefore, the usual choice is a relatively simple excitation mechanism, such as the one used in the study of the structural behavior of an electric guitar body [19]. Its sole purpose is to pluck a string with high repeatability.

The second scenario is concerned with human–instrument interaction and primarily investigates the performance of the player, including gestures and the resulting sounds. A series of recent studies applied a particularly interesting method, combining a light-payload collaborative robot arm with switches and a strain gauge as calibration devices [20], or with advanced motion tracking to analyze and mimic human bowing performance [21,22,23]. The applied setup allowed for the very flexible reproduction of human gestures and provided a large amount of acoustic and gesture data [24,25]. However, the motion capture of a musician, particularly in the case of string instruments, may be intrusive and must implement kinematic models to fill in the missing data [26]. Thus, while the captured movement parameters, such as bow position, velocity, and acceleration, seem to be reproduced with very good details, the robot cannot reproduce the sound produced by a human performer well enough [23], implying that not only the kinematics but also the dynamics of the system must be analyzed.

While collaborative robots share the same level of precision of mechanics and kinematic positioning as their industrial counterparts, the way they are programmed reproduces human inaccuracies. Unlike industrial robots, such as KUKA, ABB, and Fanuc—which have their process points set in Cartesian space and use Denavit–Hartenberg notation to calculate optimal process times—collaborative robots, like those from Universal Robotics, are different. Their process paths are set manually by an operator guiding the end effector through the desired trajectory. This method is ideal for replicating human actions but is less suitable for research aimed at eliminating human error. The industrial use of robots in manufacturing supports this. Collaborative robots are mainly used in scenarios that involve direct human worker interaction within the robot’s workspace; hence, the need for advanced force-feedback systems. Their implementation is primarily for the safety of technical personnel.

In the third type of research scenario, the goal changes from simple sounds or performance events to a complete musical performance. Instruments are coupled with robots performing actual music, and the problems studied include performance capabilities, silence of robot operation [27], or impact of the robot on the human performers in mixed human–robot ensembles [28]. Instrument excitation mechanisms applied in such scenarios do not have to be highly repeatable or precisely mimic human movement patterns. Instead, the emphasis is on the ability to reproduce actual musical structures without producing mechanism-based noise.

Robots help to solve multiple issues that occur in instrument studies, such as the insufficient repeatability of a human operator’s actions, compared to which, a robot can be a significant improvement [29], or the lack of versatility, speed, and precision typical of simple mechanisms, a problem which a robot can solve through modularity and programming [30]. However, some issues remain. If a study involves an instrument constantly fixed to a test stand, repeatability is not an issue. But if an instrument has to be removed, which can be the case when a study involves replacing one of its elements, it is almost impossible to remount it in exactly the same position and orientation without interfering with the construction of the instrument itself, which would, in turn, modify its sound. A sub-millimeter shift between a plectrum or a bow and a string can easily result in significant changes to the recorded sound [23,31]. Even if the robot itself remains fixed and can perform movements on the sub-millimeter scale, the relative positions of the remounted instrument and the robot have to be precisely re-established with the use of additional sensors.

This manuscript presents the case as described above. We designed a robot for a study of guitars and applied it to carrying out examination of multiple guitar picks. The depth of picking varied on a sub-millimeter scale, with extreme values not exceeding the string thickness. The robot was positioned using limit switches coupled with stepper motors, which provided enough accuracy to place the effector repeatably in the required position. However, after analyzing the recorded data, it became clear that the precision was not sufficient. The source of the errors was established to be the change of a pick in the effector, which must not have been done precisely enough. The selected parameters of the signals obtained are presented. In addition to gradual changes resulting from increasing plucking depth, they display differences showing that the picks were not properly adjusted. Based on these results, a method was proposed to enhance the setup by employing acoustic feedback for additional calibration. A microphone was already part of the test stand; therefore, it could be repurposed to serve as an acoustic sensor. The acoustic data allowed us to ascertain the relative position between string and pick with precision, limited only by the stepper motor.

Our research focused on the effects of design modifications and material selection on the sound production of musical instruments. These considerations are crucial for instrument makers, who need to assess whether a specific design alteration results in the desired acoustic outcome. Therefore, the goal here was not to be able to reproduce a human movement pattern or to play complex musical pieces, but to achieve high repeatability of sound reproduction despite changes being made to the measured instrument. In order to achieve this goal, it was necessary for the measurement equipment to have the ability to automatically adjust to these changing conditions, based on sound parameters. This ensured that any observed change in sound was solely attributable to the intentional alterations made to the instrument. Without acoustic feedback, none of the solutions found in the literature are able to achieve high repeatability of sound parameters under such conditions. Mechanisms such as those used in [19] have no means of automatic adjustments. Performance robots similar to those presented in [27,28], which are designed to play music, do not have auto-adjustment capabilities, and their repeatability precision is limited, as their primary design objective is not focused on this aspect. The most powerful and flexible solutions, such as the robot arms used in the research discussed in [20,21,22,23], might serve the purpose described above. However, as described in those works, their setup was aimed at different studies, concerning human movement. They did not use acoustic feedback, instead relying on visual or strain sensor data. As their authors pointed out, repeatability of sound parameters required their expansion with additional sensors.

As presented in the brief review above, other studies involving automatic excitation of string instruments have focused either on complex, elaborate systems aimed at studying human–instrument interactions or on single-purpose mechanisms applied in well-defined problems. The solution presented in this manuscript presents a different approach. Its aim is to facilitate the performing of comparative analysis of multiple instrument specimens, in order to assess the impact of the change applied to a particular instrument element or to assess the quality of the produced instruments, for the purpose of binning. It is, therefore, designed primarily for use by instrument manufacturers as a part of the production process or in large comparative acoustic studies.

The novelty of the presented approach lies in the combination of its flexibility for studying various problems and its high repeatability, with respect to the sound produced by the examined instruments. The goal is achieved by the innovative application of a Cartesian robot with acoustic feedback for calibration and precise positioning of the string-excitation element. Automatic positioning adjustments ensure that observed changes in sound are related only to changes introduced to the instrument on purpose. Acoustic feedback can be used with other mechanisms, but simple mechanisms are unable to adjust excitation parameters in a range wide enough to use them in a broader set of tests. While robot arms have the ability to reach a wide range of excitation parameters, they are not a requirement. Simpler solutions can be effectively substituted in their place. Cartesian robots are relatively inexpensive, precise, and reliable, they can be easily operated by technicians, and they allow for programming various string-excitation scenarios. A microphone is already part of the setup for the purpose of signal recording; therefore, using it to provide an acoustic feedback allows one to calibrate and position the system without additional sensors, apart from simple limit switches. The approach is explained through algorithms, with a thorough discussion on its specifics.

## 2. The Effect of a Guitar Pick’s Attack Depth on the String Vibration

The way a string is plucked in a guitar has a significant impact on the sound emitted by the instrument. The impact here, among other things, comprises the technique, dynamics, excitation point, and excitation tool used. In the case of exciting a string using picks, a different sound will be obtained, depending on the material parameters used to produce the plectrum and its geometric parameters. In the guitarist environment, it is generally assumed that a thicker pick remains in contact with the string longer, which translates into the impulse delivered to the string. The longer the impulse, the more lower frequencies and the fewer higher frequencies appear in the signal recorded from the instrument. However, depending on the material used to produce the pick, the thickness itself does not have to be so significant. From the analysis of methods used in the sound synthesis of plucked string instruments, it can be seen that among the most important parameters are the properties of the stiffness of the excitation medium. From a material perspective, depending on the stiffness of the plectrum, the attack impulse is extended or shortened. It can, therefore, be assumed that, depending on the material parameters, different contact times with the string will be obtained with similar geometric parameters of the plectrum. Simulation models of the interaction of the pick with the string used in sound synthesis use, among others, beam mechanics subject only to transverse displacements. In the beam model, the transverse displacement of the guitar pick is in accordance with the following equation [32]:

(1) $$\frac{\partial^{2}y}{\partial t^{2}}=\frac{EI\partial^{4}y}{\rho S\partial x^{4}}$$

The characteristics of Equation (1) are length *L*, cross-sectional area *S*, mass density ρ, Young’s modulus *E*, and second moment of inertia *I*. The space coordinate along the beam is denoted by *x*, and the transverse displacement by *y*(*x*, *t*).

As mentioned above, Equation (1) is applied in sound-synthesis methods based on physical modeling, and it effectively generates a convincing sound effect. However, the actual physical phenomenon is much more complex, and similar equations are not able to precisely reproduce experimental data, such as that presented in Section 2.2. Hence, using sound feedback appears to be a more effective method for verifying if the plucking action has indeed taken place.

The string-excitation process can be described in a simplified way by the following sequences:

- the string is at rest and straight;
- the pick moves downwards at a certain speed imposed by the hand;
- the pick strikes the string;
- due to friction, the string moves with the pick and hand at a given speed;
- the area of contact between the pick and the string continues to move downwards at the same speed as the pick;
- the threshold of static friction is reached;
- the string is released;
- the string vibrates freely.

In real cases, the string-excitation process is more complicated and involves additional factors: for example, a change in the angle of attack of the pick relative to the string as a result of deformation of the fingertips holding the plectrum. If human factors influencing the path of a guitar pick were eliminated, it would be possible to study the string-excitation phenomenon using a plectrum in such a way as to obtain more information about the pick–string interaction. Among other things, for this purpose, the authors used a simple Cartesian robot in the presented research.

**Figure 1:**
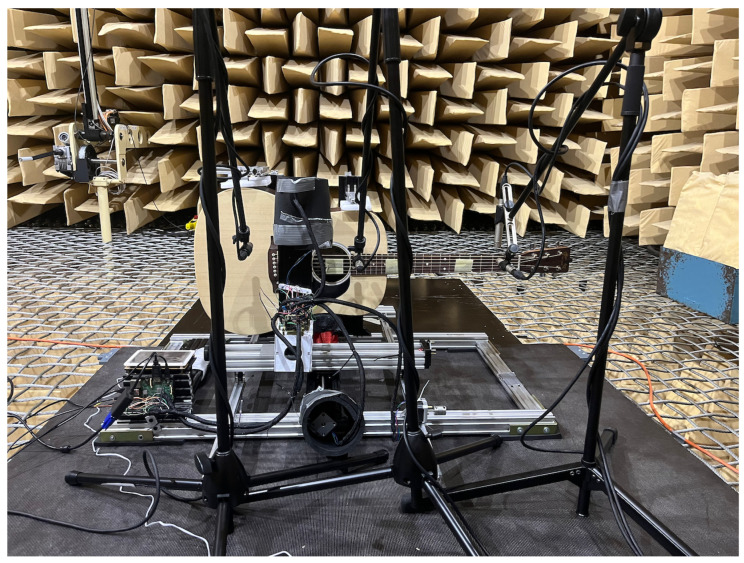
Research station equipped with a Cartesian guitar-string-excitation robot; front view.

**Figure 2:**
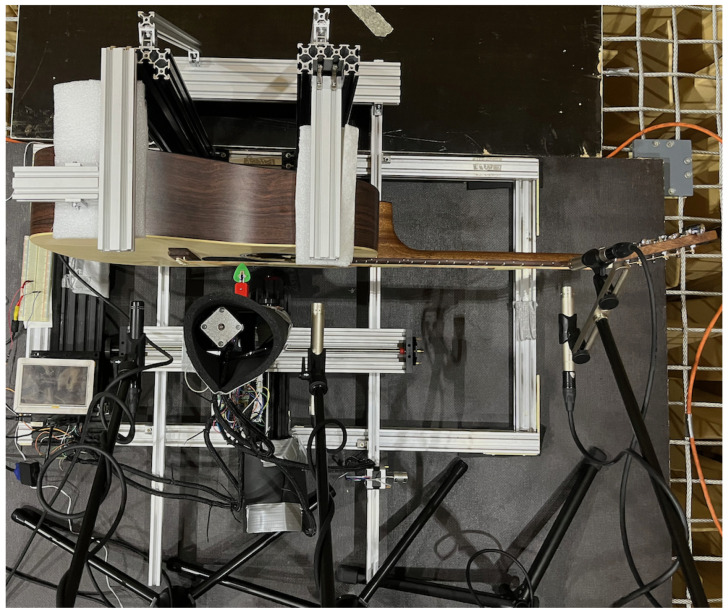
Research station equipped with a Cartesian guitar-string-excitation robot; top view.

**Figure 3:**
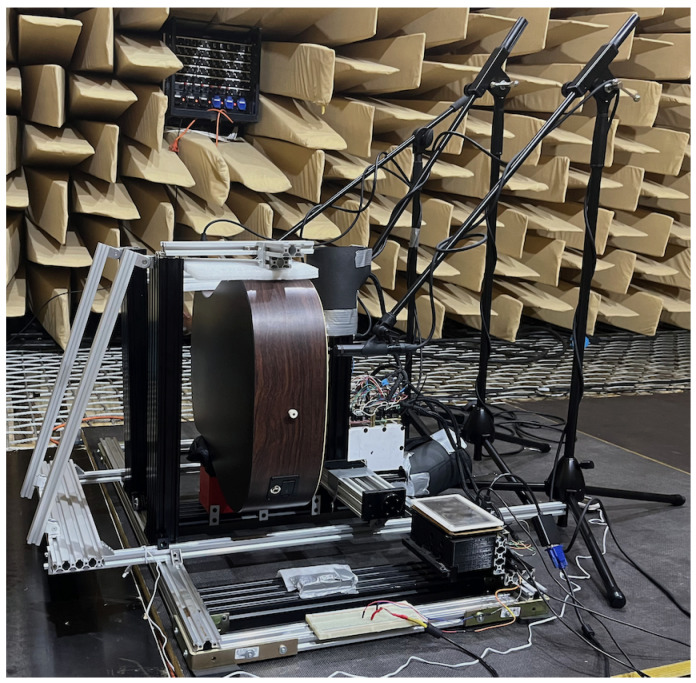
Research station equipped with a Cartesian guitar-string-excitation robot; side view.

**Figure 4:**
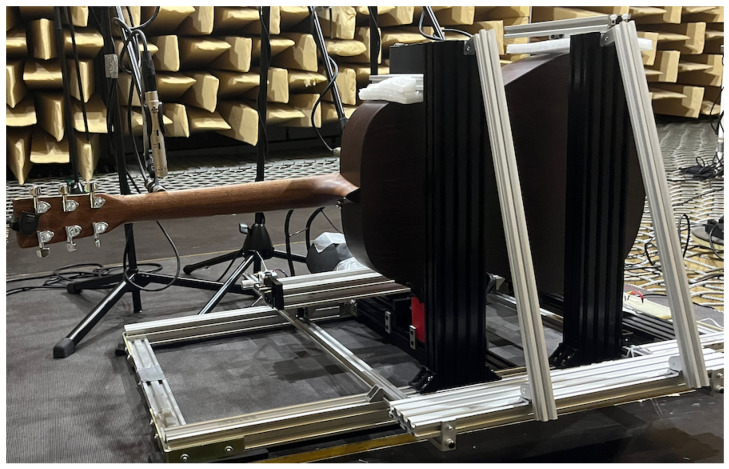
Research station equipped with a Cartesian guitar-string-excitation robot; rear view.

Our use of a robot allowed repeatable excitation of a guitar string at different depths of plectrum attack. This was used to compare various guitar plectrums and how their characteristics changed with picking depth. The experiment consisted of plucking the string with each pick, using six depth positions of the plectrum attack on the instrument string. The study was carried out in an anechoic chamber in the Department of Mechanics and Vibroacoustics of the AGH University of Krakow (Figure 1, Figure 2, Figure 3 and Figure 4).

### 2.1. Test Stand and Experiment Procedure

**Figure 5:**
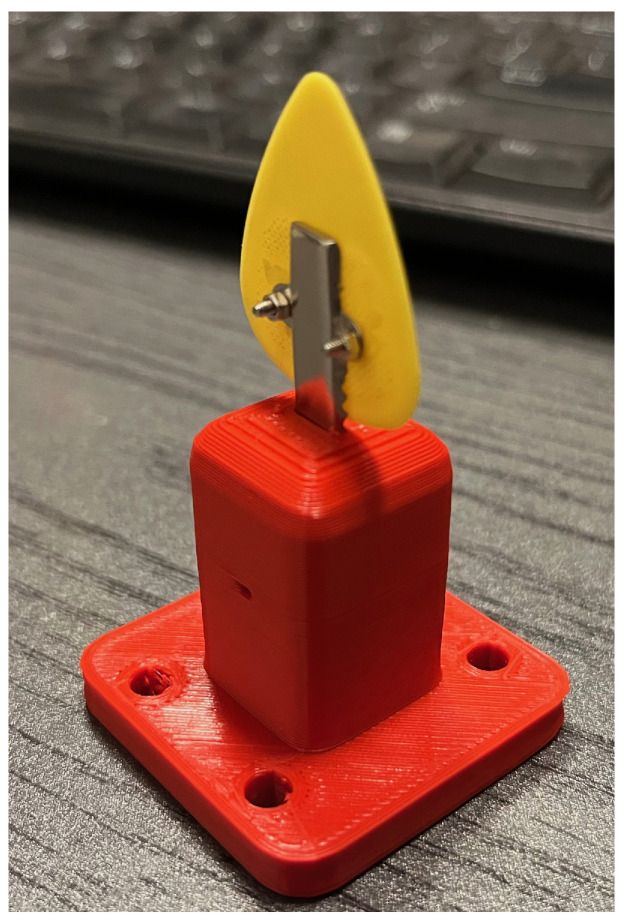
End effector for quick replacement of a plectrum. The guitar pick was mounted on a plate that could be easily removed from the mounting element.

The instrument used for this study was a Martin D-X2e electro-acoustic guitar (C. F. Martin & Company, Nazareth, PA, USA). During the experiment, guitar picks made of different materials (nylon, polycarbonate, steel, felt) were used. All of the picks, including the less popular steel and felt ones, were standard pieces obtained from music stores. They were selected to make possible the study of a wide variety of material properties and geometric parameters. To mount them on the robot, a modified end effector was used, allowing their quick replacement (Figure 5):

Condenser microphones were used to record the signal (Figure 4). A RODE MP5 (Rode Microphones, Sydney, Australia) was pointed at the lower part of the body (120 mm from the surface of the top plate), in front of the guitar bridge, and a Studio Project C4 (PMI Audio Group, Gardena, CA, USA) was placed at the height of the last fret of the instrument (120 mm from the string axis). Two more microphones (Studio Project C4 recorded the signal from the strings at the beginning of the fretboard near the second fret (35 mm from the string axis). One of them was directed perpendicularly at the surface of the neck, aiming at the fret, the other at an angle of 90° to the first one from the top of the neck. In addition, the signal from the piezoelectric transducer with which the guitar was equipped was also recorded. The signals were recorded using an audio interface (Zoom F8n Pro (Zoom Corporation, Tokyo, Japan) connected to a computer.

**Figure 6:**
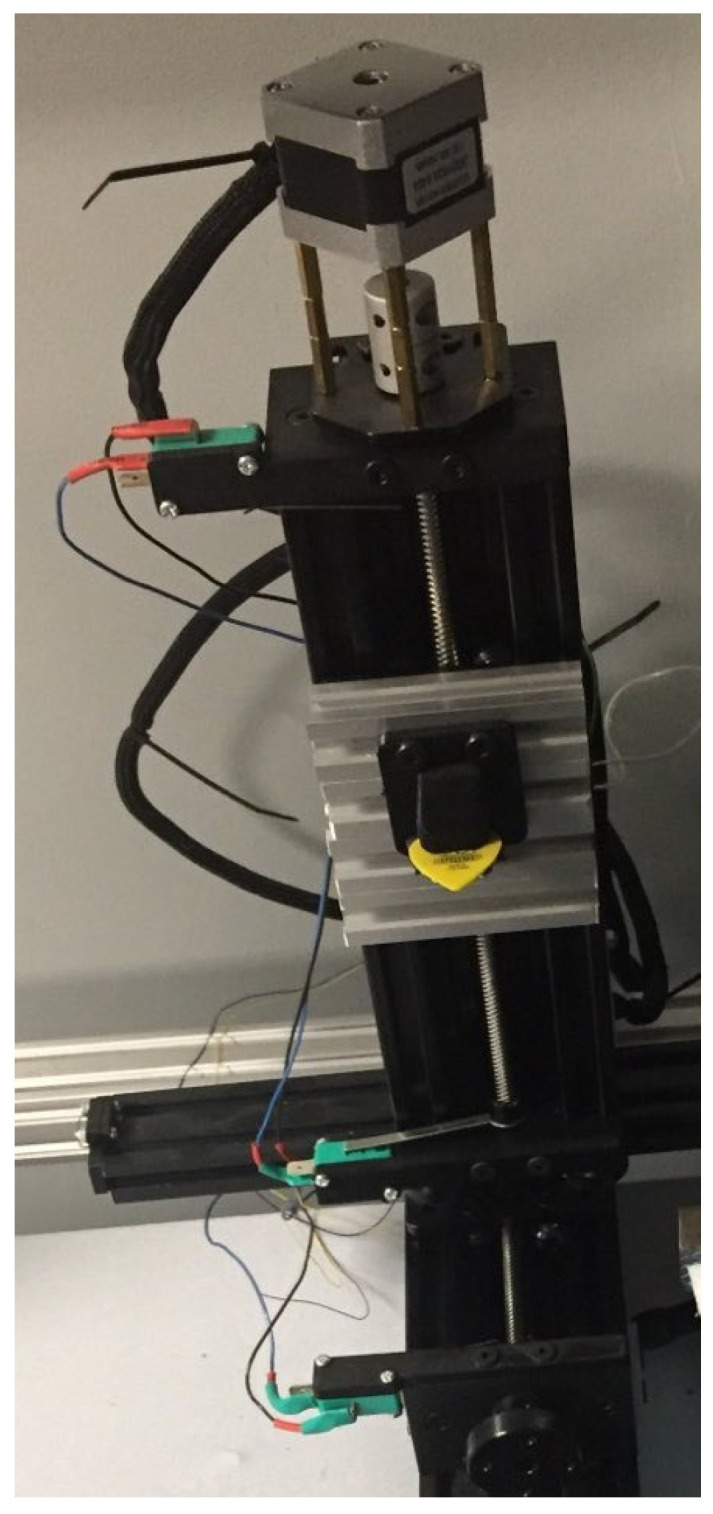
Linear actuators of the robot with limit switches at the edges of the working sections.

The robot used in the research was designed and constructed mechanically, according to the standards used in three-axis mechanisms [33,34,35,36]. The linear drives were based on stepper motors directly connected by mechanical clutches to trapezoidal screws (Figure 6). This system was used to move the carriage on which the next linear element was mounted. An additional advantage of using the mechanism was the ability to set a constant TCP (Tool Central Point—the point used to move the robot to a Cartesian position) movement speed. The motor carried out one step every $t_{stp}=0.002$ s, while the thread pitch was $P_{h}=8$ mm. To determine the linear displacement velocity of the TCP in the *z*-axis, the following formula was used:

(2) $$v_{tcp}=\frac{Ph}{200\cdot t_{stp}}=\frac{8}{0.4}=20\;\left[\frac{\mathrm{mm}}{s}\right].$$

The TCP used to determine the tool orientation and position in Cartesian space was set at the pluck point of each individual guitar pick used in the experiment.

Limit switches were used to determine the extreme points of the axis. To determine the current position relative to the given coordinate system, the control software used the coupling of information from the limit switches and the number of steps performed by the motor. The position of the printing tip in modern 3D printers is determined by a similar principle.

**Figure 7:**
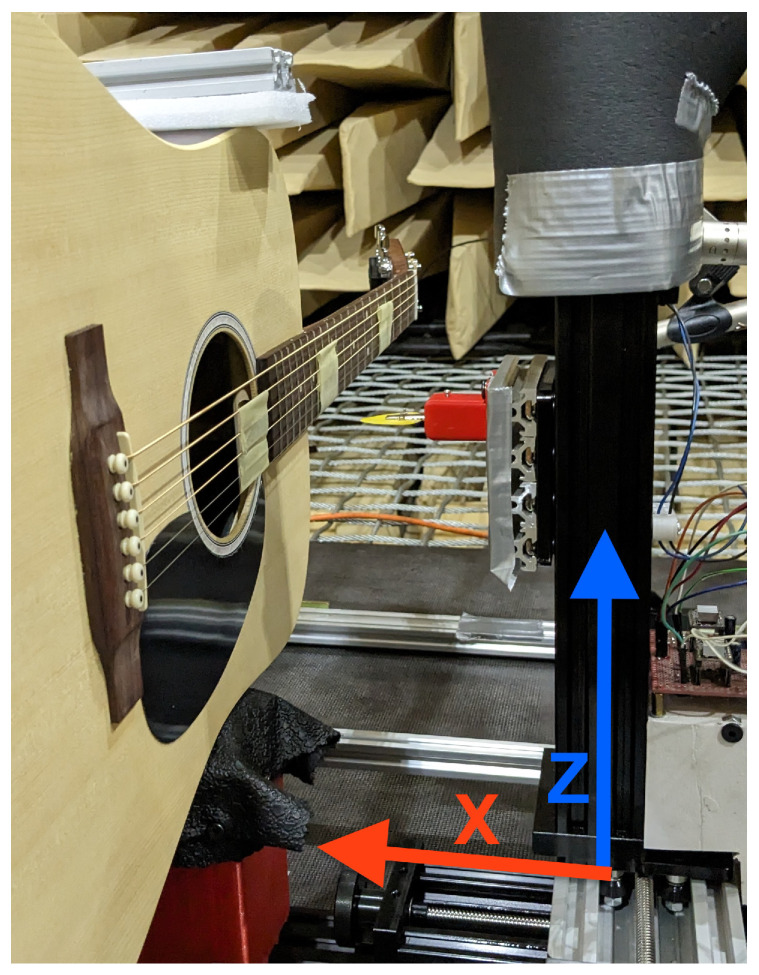
Experimental setup: instrument and plucking robot with axes overlayed.

In the experiment, the sixth string of a guitar tuned to the note $E_{2}$ ($f_{0}=82.41$ Hz) was excited (Figure 7). In order to standardize the coordinates of the position of the end effector used in the routines relative to the TCP (pluck point), the differences in the length of the individual picks were compensated for by the length of the mounting plate connecting the end effector with the pick and ensuring a quick exchange of the plectrum (Figure 5). During the test, a constant ambient temperature of 19 °C was maintained.

**Figure 8:**
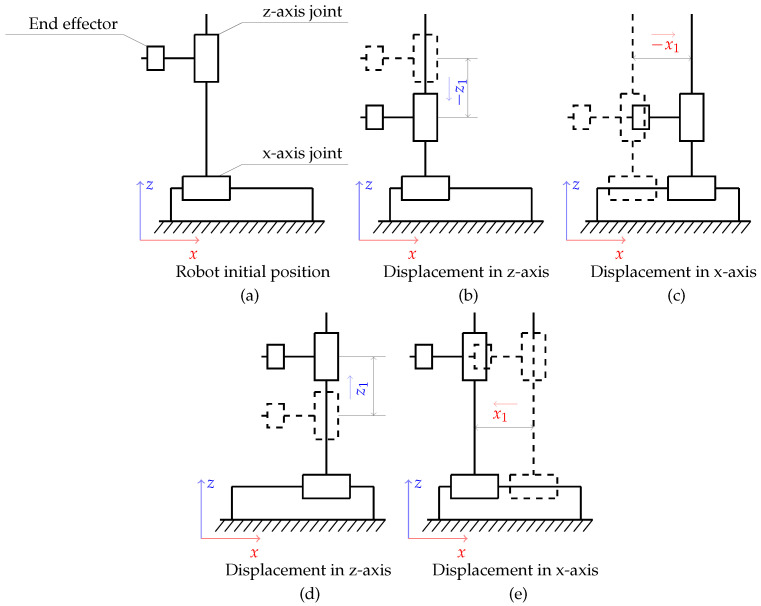
Simplified diagram of the robot movement, with $z_{1}$—displacement along the *z*-axis—and $x_{1}$—displacement along the *x*-axis; (**a**–**e**) show the subsequent phases of the movement.

In order to excite the string, the robot performed the movement as in the reference figure (Figure 8). Figure 8a shows a simplified diagram of the robot used in the study. The numbers 1, 2, and 3 indicate the individual joints of the manipulator. The robot performed movement along two axes, the *x*-axis and the *z*-axis, in the process of exciting the string. Movement along the *z*-axis caused the pick to attack the guitar string and its return to the initial position; movement along the *x*-axis was used to bypass the string while the end effector returned to the starting position. The movement scheme was dictated by the need to excite the string from the same direction, and it proceeded as follows:

1. displacement by distance $-z_{1}$ (Figure 8b) and excitation of the string in half of $z_{1}$;
2. (after t=30 s) displacement by distance $-x_{1}$ (Figure 8c);
3. displacement by distance $+z_{1}$ (Figure 8d);
4. displacement by distance $+x_{1}$ (Figure 8e).

The sign representing the direction of displacement along *x*-axis (for $x_{1}$) was determined by the experimental setup presented in Figure 7.

**Figure 9:**
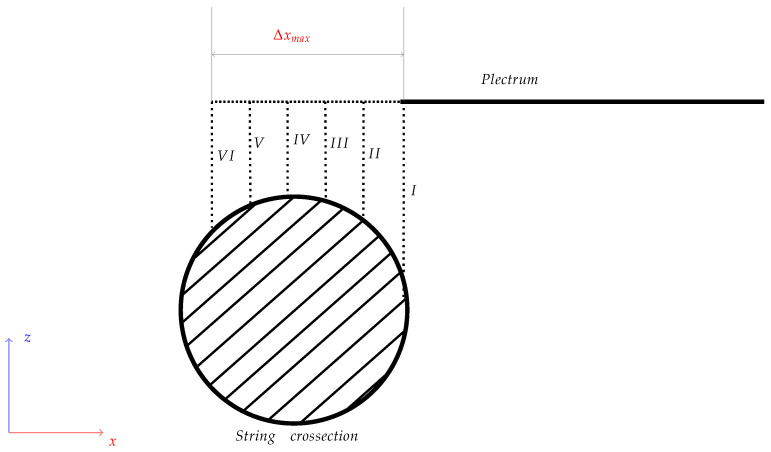
Simplified diagram showing the string-excitation points on string cross-section during the test. Roman numerals I–VI indicate subsequent positions of the plectrum displacement; $\Delta x_{max}$ is the range of TCP movement on the *x*-axis and was equal to 0.96 mm. The thickness of the tested string was 1.4224 mm.

Additionally, after performing ten plucks, the position of the string-excitation point was changed. The robot moved towards the guitar body, increasing the depth of the plectrum relative to the string axis by 160 µm (Figure 9). After performing ten excitations for each of the six positions, the tested guitar pick was exchanged and the whole procedure was repeated. The first excitation position was the edge of the string; the last, the sixth point, was about 0.96 mm in the cross-section of the string.

### 2.2. Experimental Results

The obtained results were segmented into individual plucks, which were then analyzed. A set of acoustic signal parameters [37,38,39] was calculated and averaged within the measurement series (https://github.com/Jasinsk/InstrumentAnalyzer accessed on 1 March 2025). The spectrum centroid was calculated using the following formula:

(3) $$SC=\frac{\sum_{n=1}^{N}n\cdot S\left(n\right)}{\sum_{n=1}^{n}S\left(n\right)},$$

where *n* is the signal spectrum bin iterator and S(n) is the signal amplitude for *n*-th bin.

**Figure 10:**
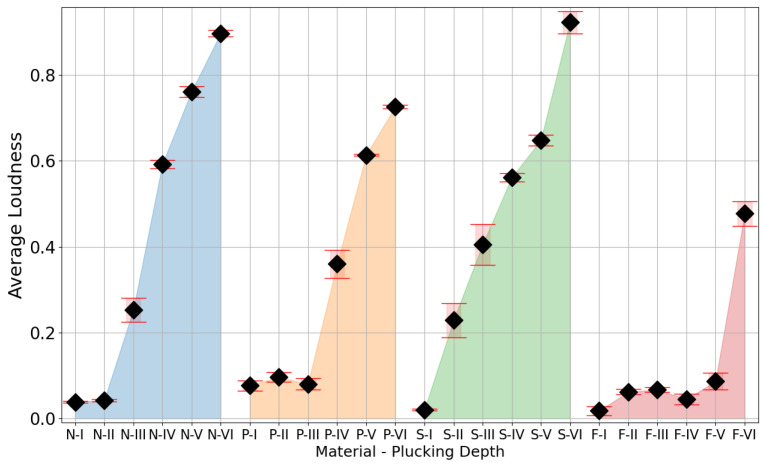
Comparison of average relative loudness values, calculated according to ISO 532-1:2017 §6 [40], with standard deviations for different material and plucking-depth configurations. Capital letters denote plectrum material: N—Nylon, P—Polycarbonate, S—Steel, F—Felt. Roman numerals denote subsequent depths of the plectrum during plucking.

**Figure 11:**
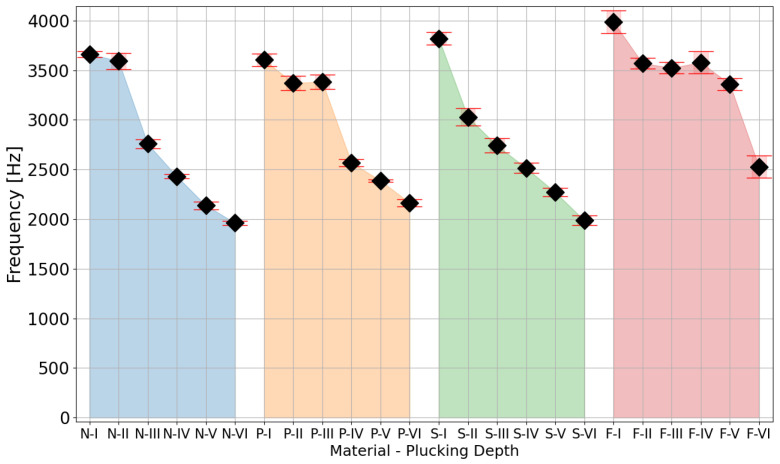
Comparison of spectrum centroid values with standard deviations for different material and plucking-depth configurations. Arabic numbers denote plectrum material: N—Nylon, P—Polycarbonate, S—Steel, F—Felt. Roman numerals denote subsequent depths of the plectrum during plucking.

The preliminary analysis of the values of the signal loudness parameter (Figure 10) shows a monotonic increase in the value with the increase in the depth of the plectrum relative to the axis of the test string. These results show the dependence of this parameter on the dynamics of playing the instrument. Additionally, increasing the depth of the plectrum attack point on the string reduced the frequency of the signal’s spectrum centroid, which indicated an increase in energy in the lower-frequency bands of the signals (Figure 11).

The study showed how small changes in the position of the plectrum attack on the guitar string affect the signal obtained. The effective values obtained of the signal for individual positions of the attack on the string show an increase in the loudness parameter with the increase in the depth of the plectrum relative to the cross-sectional area of the string. In the described study, it was necessary to use a robot that allowed precise displacement of the plectrum for small distances (in this case, 160 µm). The use of a robot equipped with linear actuators maintaining the position guaranteed the set depth of the pick attack on the instrument string. The results obtained also showed differences depending on the plectrum used.

**Figure 12:**
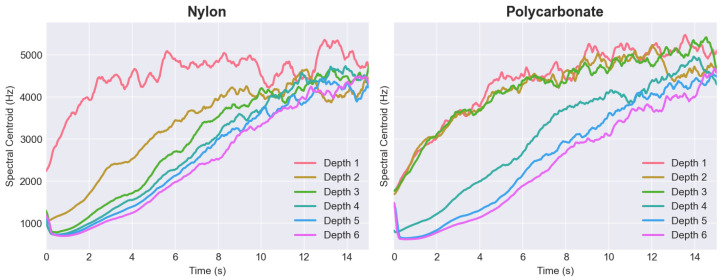
Comparison of spectrum centroid values in time for nylon and polycarbonate plectrums at different plucking-depth configurations.

The analysis of the results indicates that for all the materials, except for steel, a ledge appeared at shallower plucking depths. In this specific range, we observed that neither the loudness nor the timbre significantly changed with an increase in plucking depth. This implies that, regardless of the precision in plectrum positioning, alterations in plectrum shape and material influence the depth required for optimal string excitation. Figure 12 illustrates the variation of the spectral centroid over time when the string was plucked at various depths, using both nylon and polycarbonate plectrums. These results show how at a low plucking depth, despite a pluck being visible and registrable, its characteristic drastically deviated from the shape visible for further depths. As the depth of the pluck increased, the graphs demonstrate a decrease in the differences between successive plucks and a growing similarity in their shapes. The outcomes for depth 1 in nylon and depths 1–3 in polycarbonate deviated from a standard decay curve. Therefore, these should not be evaluated as legitimate plucks, as musicians would not categorize them as such.

The results of this study of plucked string instruments lead to two clear conclusions. Firstly, it is important to note that if a string is plucked below a certain depth threshold, the sound produced may not accurately reflect the instrument’s true timbre when played more typically by a musician. Secondly, the variations in mechanics between different plucking mechanisms and plectrums are significant enough to make it impossible to suggest a purely geometric universal plucking approach.

## 3. Using Acoustic Events for Calibration of the Effector Position

Using limit switches coupled with stepper motors allowed the robot to position the effector with a step of 40 µm. This mechanism was applied in the study presented in the previous section, to designate a work area for the effector movements safe for the studied instrument. However, the geometric irregularities of a guitar pick and the instrument itself prevented the precise repositioning of the pick in relation to the string after each replacement, even with additional adjustments. This can be observed as a flat area of loudness values in Figure 10 and spectrum centroid values in Figure 11, where movement of the effector did not evoke the corresponding change in the acoustic parameter. Clearly, a purely geometric repositioning based on stepper motors and limit switches was not sufficient.

Therefore, we developed a method to utilize the microphones already present in the test stand as acoustic sensors and the registered sound events for positioning. The method uses two kinds of sound events: qualitative and quantitative. The former is the presence of the sound of a plucked string. The latter is the change in the delta values between the observed acoustic parameters.

**Figure 13:**
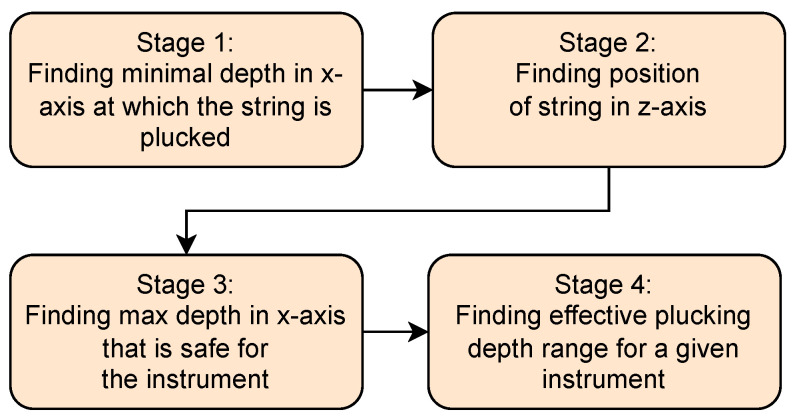
Diagram showing all stages of the algorithm.

The procedure is divided into four stages (Figure 13):

1. Establishing the beginning of a plucking range, which is the first position of the effector on the *x*-axis that produces any plucking sound;
2. Establishing the precise position of a string along the *z*-axis;
3. Establishing the end of a plucking range, which is the last position of the effector on the *x*-axis that allows for plucking a string without a risk of breaking it;
4. Establishing the beginning of a useful plucking range, which is the position of the effector on the *x*-axis where a measured acoustic value begins to change significantly.

**Figure 14:**
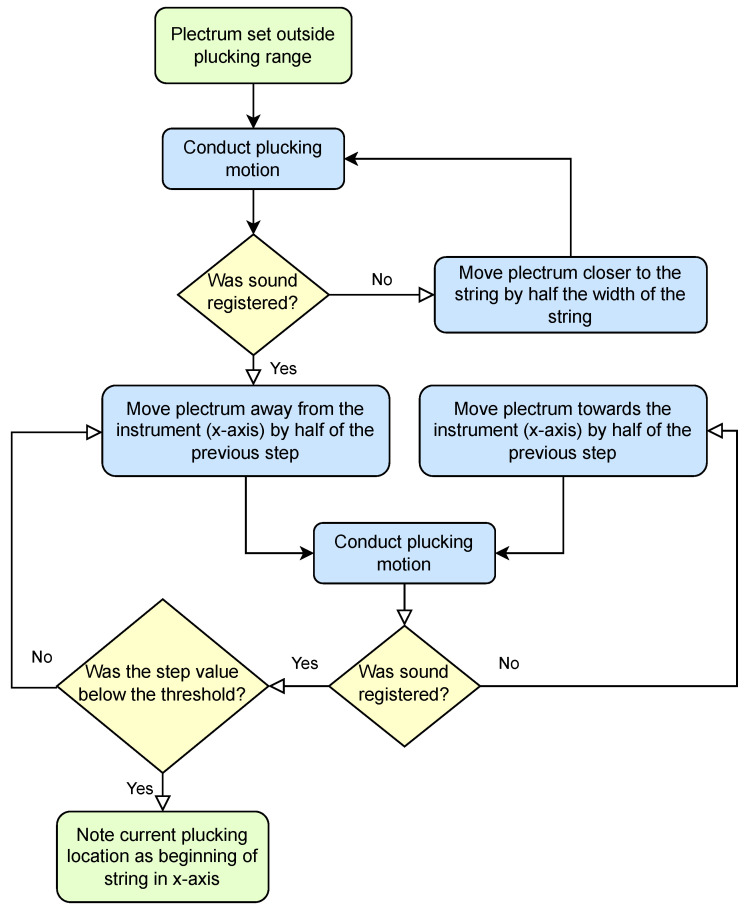
Stage 1: Establishing the beginning of a plucking range.

The first stage (Figure 14) sets the plucking element anywhere outside the plucking range, which can be done without any acoustic aid, on the sole basis of limit switches and stepper motors. Then, the plucking element attempts a plucking movement, and the sound sensor observes the result. The movement is repeated with the plucking element shifted towards a string. Once the first pluck has been registered, a halving procedure is applied, to shift the plucking element around the minimum depth at which plucking occurs in decreasing steps, so as to establish its position with the highest precision allowed by the stepper motor.

**Figure 15:**
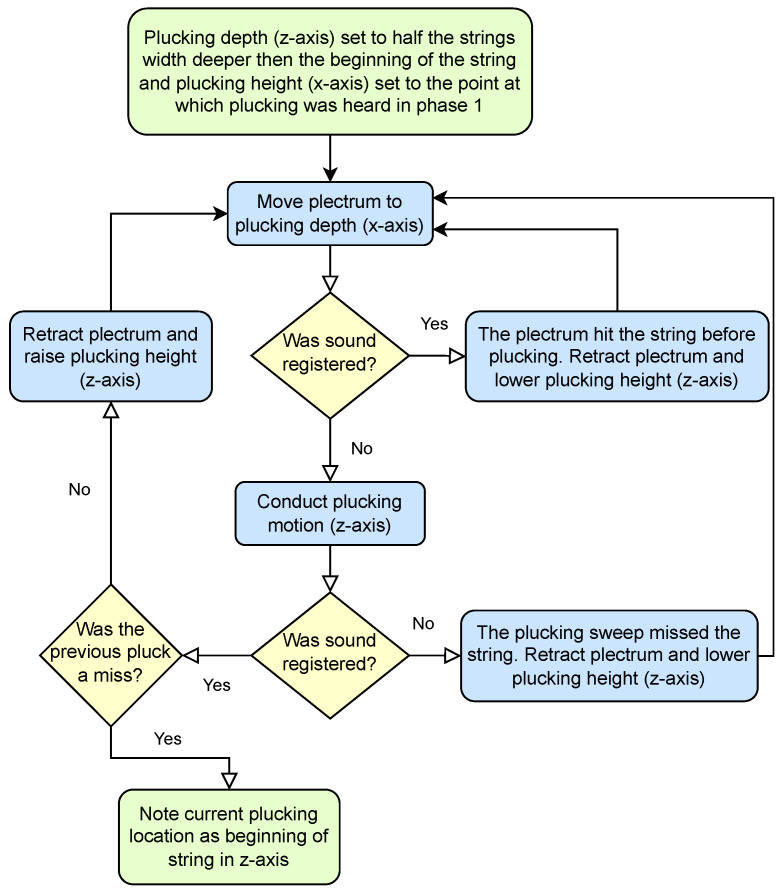
Stage 2: Establishing precise position of a string along the *z*-axis.

The second stage (Figure 15) starts from the plucking position found in the first stage. It attempts to establish the precise position of a string edge along the *z*-axis in a series of plucking movements with increasing range along the *z*-axis. A sound can be produced not only by plucking, but also by hitting the string while moving along the *x*-axis to the plucking position. Both of these sound events are recognized and used in the algorithm.

**Figure 16:**
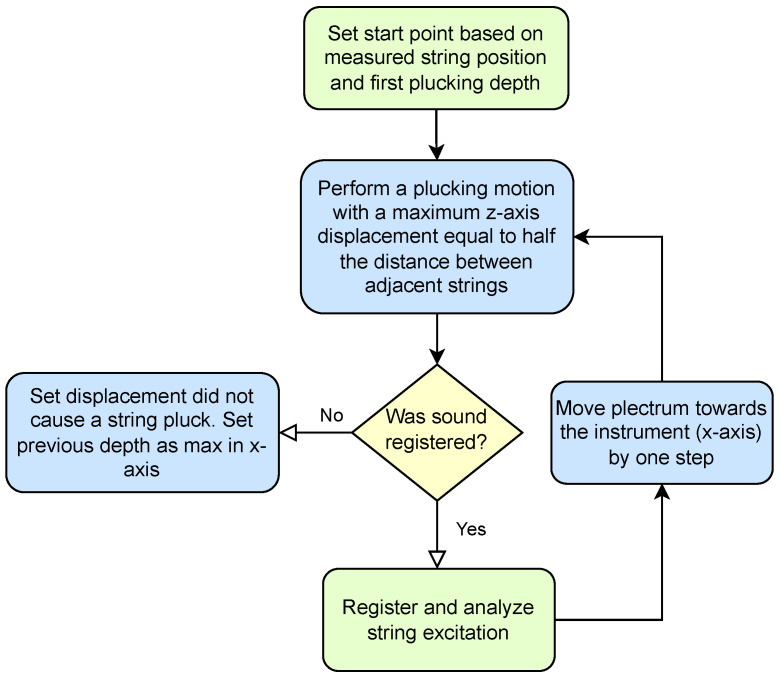
Stage 3: Establishing the end of a plucking range.

If a string is excited by plucking, the maximum amplitude of the string vibrations depends on the initial displacement. The displacement is obtained by straining a string with a plectrum. Under ideal conditions, when no striking of a string occurs, the amplitude depends only on the point where the string detaches from the plectrum. In this case, the velocity of the plectrum does not play a significant role. Larger displacements are obtained by moving the plectrum towards the instrument and straining the string with increasing area. However, there is a material limit that corresponds to breaking the string, which must be considered when programming robot movements. Therefore, movement along the *z*-axis is limited to a given known value. The third stage (Figure 16) finds the position of a plectrum on the *x*-axis, which corresponds to the last safe string displacement. The robot attempts to pluck the string with a pick displacement along the *z*-axis, set to the maximum safe amplitude. After each pluck, the plectrum is moved towards the instrument. If the plucking movement does not produce sound, this means that the given string displacement required to produce the pluck would exceed the point of maximum safe plucking, and, thus, the end of the plucking range is established.

**Figure 17:**
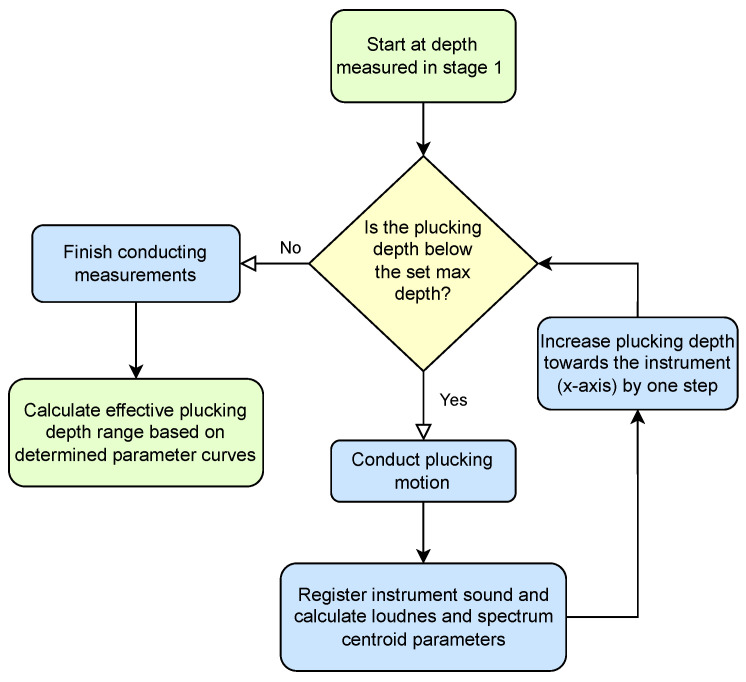
Stage 4: Establishing the beginning of a useful plucking range.

**Figure 18:**
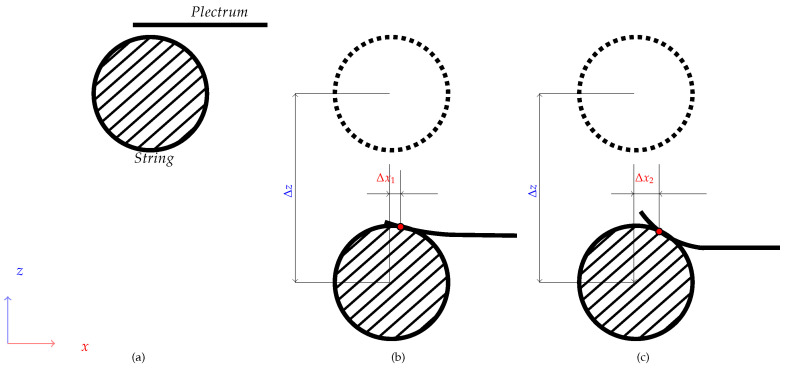
Simplified diagram showing the excitation points of the string on its cross-section, depending on the elasticity of the pick. Subfigure (**a**) presents the pick before the pluck; (**b**) presents displacement of the pluck point for a more rigid plectrum; (**c**) presents displacement of the pluck point for an elastic plectrum; $\Delta x_{1}$ and $\Delta x_{2}$—displacements of the pluck point in relation to the center of the string; Δz—displacement of the string from its equilibrium position.

The final stage (Figure 17) was not required to carry out the study, but it allows for limiting the work area, to examine only scenarios that provide meaningful acoustic data. This can also be seen as the establishment of a common point of reference based not on spatial but on acoustic conditions. At this stage, the plucking range established in the previous stages is examined, and selected parameters, such as loudness or spectral centroid, are observed with increasing plucking depth. If the parameter delta between two subsequent steps exceeds the assumed minimum then the depth of the respective displacement is considered to be the beginning of a useful plucking range. In the case of the study presented in Section 2, the elastic deformation of the guitar pick (shown in Figure 18) prevented the string from attaining larger displacements in some of the initial steps. This phenomenon occurs for elastic picks but not for a steel picks, as can be observed in the parameter values presented in Figure 10 and Figure 11, proving the merits of the range adjustment procedure. It is worth noting that the fourth stage can be carried out in parallel with stage three.

The aim of detailing the algorithm is to demonstrate the precise method of incorporating acoustic feedback into the control mechanisms of a Cartesian robot. This integration enables the robot to automatically modify the pick position in relation to the instrument. The use of qualitative sound events helps establish an effective excitation zone. Quantitative events help refine this zone to an area that yields only significant acoustic data.

Although it has been discussed for the case of a guitar, the procedure can be easily adapted to other string instruments studied using Cartesian robots and excited not only by plucking but also by striking or bowing.

## 4. Discussion

The experimental results presented in Section 2 (Figure 10 and Figure 11) show that even minimal changes in the attack depth (160 µm) of the guitar pick on the string noticeably affected the parameters of the acoustic signal produced by the instrument. This shows the need for a repeatable instrument-mounting system and a precise positioning system.

However, a system based solely on geometry proved inaccurate, despite the robot positioning system’s high precision. The main issue originated from the positioning of the instrument and its components in relation to the robot effector. This will always be a problem with acoustic instruments that do not allow a firm and repeatable grip during the study. Even small displacements can create measurable changes in the signal, indicating the need for additional sensors to enhance the existing feedback system. Considering the difficulties in maintaining the position of the instrument after its disassembly from the stand, as mentioned in the previous sections, a calibration algorithm was proposed, using an acoustic sensor observing two types of acoustic events. This method established a common and reliable acoustic reference point, allowing for comparison across the various cases studied.

Acoustic feedback is a simple and efficient way to significantly enhance positioning precision. However, the method has some limitations. The use of sound requires a controlled acoustic environment. Environmental noise or random sound events can affect the calibration process. The environment is relatively easy to control under laboratory conditions, but in manufacturing facilities it can require additional measurements and adjustments. Another issue is the noise generated by the robot. Several approaches can be used to suppress it, but it will still be a part of the recorded signal, requiring proper signal processing, possibly adjusted to take into account both the robot and the environment. Finally, while this manuscript discusses a case of studying guitar picks, the calibration procedure might need to be adjusted for other scenarios.

## 5. Conclusions

In our study, we have introduced a novel method of automatic position adjustment of the excitation element in relation to the musical instrument. Unlike the methods presented in the literature, which are either solely based on geometry or incorporate visual feedback, our method uses sound events registered by a microphone, to establish the area in which the movement of a Cartesian robot produces sound. This allows for the selection of the study area, so as to ensure that the sound parameters are comparable despite modifications introduced to various elements of the instrument. As a consequence, any observed changes in sound are solely attributable to the intentional alterations made to the instrument, allowing for study of their effects. Unlike other methods, our solution utilizes acoustic feedback to consistently maintain sound parameters even under varied conditions.

Our manuscript describes a particular use case of the proposed method, where limit switches and stepper motors were supplemented with acoustic sensors in a Cartesian robot designed for use in guitar testing. This setup allowed us to precisely adjust the work area based on acoustic factors. A study was presented, in which the robot was used to precisely pluck a string with several types of plectrum, made of various materials, to observe the impact of each plectrum on the quality of the sound produced. The robot allowed for the plucking of a string with varying amplitudes controlled by increasing the depth of the plectrum in steps of 160 µm. The study found that, despite the robot’s ability to consistently position the effector as required, maintaining the relative positions of the robot and the instrument or its components presented a challenge when certain elements needed replacement during the study. This led to the conclusion that positioning the effector only on the basis of geometry is insufficient, and that the proposed procedure, which incorporates acoustic sensors, is necessary. For acoustic measurements, sensors are already installed on the test stand as microphones to record sound data. They can be repurposed to monitor specific acoustic events that guide positioning adjustments. The procedure allows for the establishment of a common acoustic reference point in measurements in which the qualities of the sound signal are to be studied. The procedure can introduce adjustments to effector positions, to take into account phenomena such as elastic displacement of a plectrum, as shown in the graphs obtained from the measurements. Details of the entire procedure have been presented in the form of algorithms, and it can be adapted for the needs of different string instruments.

The proposed method does not require additional equipment; thus, it can be implemented through additional programming of the robot alone. This may not only minimize deviations in signal parameters related to the accuracy of the instrument mount at the test stand but may also allow for the extension of its functionality, in terms of controlling the dynamics of the guitar string excitation. The approach combines relative simplicity of application, flexibility, and high repeatability with respect to the sound produced by the instruments examined. Instrument manufacturers can apply it as part of the production process, to assess the quality of instruments or study the impact of changes introduced to their design. An additional advantage is the potential for versatility in implementation. The proposed algorithm can be used to calibrate the device live during the ongoing experiment, in order to normalize the results obtained. This could significantly shorten the process of analyzing the data obtained in the experiments, reducing the amount of deviated signals. In addition, the algorithm described can allow for planning and implementation of more advanced experiments with string instruments that are impossible to perform without robotic support.

## Data Availability

The data presented in this study are available upon request from the authors.
